# The association between mRNA expression of resistin, TNF- α, IL-6, IL-8, and ER-α in peripheral blood mononuclear cells and breast cancer

**DOI:** 10.3906/sag-2008-292

**Published:** 2021-06-28

**Authors:** Maasoumeh ZARE MOAIEDI, Fatemeh AHMADPOOR, Mojtaba RASHIDI, Ahmad AHMADZADEH, Amir Ahmad SALMASI, Ghorban MOHAMMADZADEH

**Affiliations:** 1 Department of Clinical Biochemistry, Faculty of Medicine, Ahvaz Jundishapur University of Medical Sciences, Ahvaz Iran; 2 Department of Hematology-Oncology, Faculty of Medicine, Firoozgar Clinical Research Development Center, Iran University of Medical Sciences, Tehran Iran; 3 Department of Surgery, Faculty of Medicine, Ahvaz Jundishapur University of Medical Sciences, Ahvaz Iran; 4 Department of Clinical Biochemistry, Faculty of Medicine, Ahvaz Jundishapur University of Medical Science, Hyperlipidemia Research center, Ahvaz Iran

**Keywords:** Breast cancer, resistin, tumor necrosis factor-alpha, interleukin-6, interleukin-8, estrogen receptor alpha

## Abstract

**Background/aim:**

Adipocytokines, adipose tissue-derived proteins, were demonstrated to be involved in the pathogenesis of breast cancer. We assessed the mRNA expression of resistin, tumor necrosis factor-alpha (TNF-α), interleukins 6 and 8 (IL-6, and IL-8), and estrogen receptor alpha (ER-α) in peripheral blood mononuclear cells **(** PBMCs) of women with and without breast cancer.

**Materials and methods:**

The PBMCs were isolated from the whole blood of 32 women with breast cancer and 18 women without breast cancer using density gradient centrifugation. The mRNA expression of the target genes was measured by reverse-transcription polymerase chain reaction (RT-PCR). Body mass index was calculated, additionally, clinicopathological characteristics of the breast cancer patients were determined by histopathological examination.

**Results:**

The mRNA expression of resistin (3.5-fold) and IL-6 (15-fold) in PBMCs of breast cancer patients significantly increased in comparison to healthy controls. Resistin expression was significantly associated with inflammatory markers including TNF-α, IL-6, IL-8, but not with anthropometric indices. Logistic regression analysis revealed the studied adipokines were not associated with breast cancer. Based on the ROC curve analysis the diagnostic performance of IL-6 was significant (0.825, 95% CI: 0.549-0.94, p = 0.030), thus, it might be considered as a breast cancer biomarker that reflecting an early and inflammatory stage of the disease.

**Conclusion:**

Breast cancer is not associated with increased expression of inflammatory cytokines in PBMCs. Our results suggested that a PBMC-based gene expression test may be developed to detect breast cancer early.

## 1.Introduction

 Breast cancer, the most common malignancy in women worldwide, recently, considered a major global health burden in all nations [1]. Previously, a considerable and potential role of adipokines in the development of breast cancer has been suggested [2]. Resistin, a 12.5 kDa peptide, was first detected in mouse adipocytes where has been associated with insulin resistance and the development of type 2 diabetes [3]. Several studies found that human serum resistin was associated with different cancers including breast, prostatic, colorectal, and endometrial cancers [4-7]. 

Tumor necrosis factor-alpha (TNF-α), a pro-inflammatory cytokine, was originally identified in adipose-tissue infiltrated-macrophages, and its serum concentration was positively associated with body mass index (BMI) [8]. In overweight and obese individual’s clinical trials, it has been reported that an increased level of TNF-α expression was associated with the increased risk of breast cancer [9]. 

Interleukin-6 (IL-6), a pleiotropic cytokine, was originally produced by different cell types such as endothelial and normal hematopoietic cells [10]. It is an effective pleiotropic cytokine that is considered a key growth-promoting and anti-apoptotic factor [11]. In breast tumors, the major sources of IL-6 are including tumor-derived fibroblasts, and tumor-infiltrated macrophages and lymphocytes [12]. However, IL-6 activity in cancer has not been completely understood, and its controversial roles in both tumorigenesis and tumor suppression have been reported [13]. 

Interleukin 8 (IL-8), well-known as a chemotactic cytokine, is the main factor for the recruitment of neutrophils and causes the initiation of an inflammatory response [14]. Several studies demonstrated that the expression of IL-8 increased in several human tumors [15,16] and was usually accompanied by malignancy [17]. The effect of IL-8 on the migration and invasion of tumor cells is vital for the development and cancer metastasis [18]. Some studies have been demonstrated that monocytes, T cells, and B cells could respond to estrogens, and the increased level of estrogen receptor alpha (ER-α) and estrogen receptor beta (ER-β) expression in these cells have been reported [19,20]. We investigated whether the mRNA expression of resistin, TNF- α, IL-6, IL-8, and ER-α in PBMCs are associated with breast cancer. 

## 2.Materials and methods 

### 2.1. Study populations

The study population that participated in the current research was 40 Iranian females. Breast cancer patients ( *n* = 32) were attended at Ahvaz Golestan Hospital, Ahvaz, Iran, from January 2018 till November 2019. Of the 32 women diagnosed with cancer, 16 women were new cases without any treatment, 8 women were recruited before receiving initial adjuvant chemotherapy (chemotherapy after surgery), and 8 women were recruited before receiving neoadjuvant chemotherapy (chemotherapy before surgery). Blood samples were obtained before the participants’ first chemotherapy. Adjuvant chemotherapy patients were those who were at least in their 4th-week postsurgery (lumpectomy or mastectomy). The clinical diagnosis of breast cancer was confirmed by the histopathological examination of the tumor tissue samples. Hormone receptor status was checked according to the medical records with cytopathology or immunohistochemistry. Demographic characteristics including age, height, weight, and BMI were measured by the same physician under similar conditions at the same time. Breast cancer patients were further divided into two subgroups: those without surgery (n = 16) were new cases and confirmed from the sample obtained by biopsy, and those with surgery who were diagnosed and confirmed with histopathological examination of the tumor tissue samples. Healthy control individuals ( *n* = 18) were age and BMI matched to breast cancer patients. The healthy control group was randomLy selected from the Outpatient Clinic of Internal Medicine at the same hospital among women who came for an annual check-up examination and given a negative mammogram result showing the absence of breast cancer and other malignant breast diseases. All subjects enrolled in the current study were Iranians with the ancestry of Khuzestan Province, southwest of Iran, and all of them gave written informed consent. The study protocol was approved by the Ethics Committee of the Ahvaz Jundishapur University of Medical Sciences (IR.AJUMS.MEDICINE.REC.1398.005).

### 2.2. Sampling and PBMCs isolation

The venous blood sample was collected from preoperative patients before starting treatment. Another set of blood samples was also collected from 18 healthy control women. The density gradient centrifugation by Ficoll-Paque (Pharmacia, Freiburg, Germany) was used for PBMCs isolation. First, 3.5 mL of Ficoll was transferred into a centrifugation tube. Then, anticoagulated blood samples were diluted with a 1:1 ratio with phosphate-buffered saline (PBS, pH = 7.4), and cautiously pipetted over a Ficoll-Paque gradient (7 mL/tube). The samples were centrifuged at 600 × g for 20 min at room temperature. The PBMCs containing layer was removed and the cells were washed two times with PBS, pH = 7.4. Finally, the isolated PBMCs were stored at −80°C until used for RNA isolation.

### DNA synthesis and real-time polymerase chain reaction (PCR) analysis 2.3. RNA extraction, c.DNA synthesis and real-time polymerase chain reaction (PCR) analysis 

**Figure 1:**
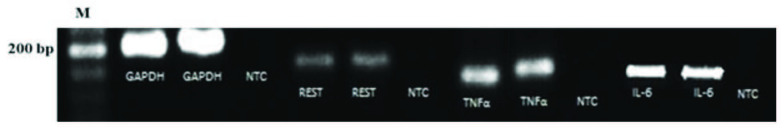
The pattern of PCR products was amplified by GAPDH, Resistin, TNF-α, IL-6, IL-8, and ER-α specific primers. Lane M: 50 bp ladder (Fermentas, Germany), Lane 2: GAPDH product (194 bp); Lane 3: Nontemplate control (NTC); Lane 4: Resistin product (119 bp); Lane 5: NTC; Lane 6: TNF-α product (102 bp); Lane 7: NTC; Lane 8: IL-6 product (164 bp); Lane 9: NTC; lane 10; IL-8 product (68 bp); lane 11: NTC; Lane 12: ER-α product (85 bp); Lane 13: NTC.

Total RNA was extracted from PBMCs using Favor Pre Total RNA mini kit (Favorgen Biotech Corp. Wembley, Australia). The concentration of RNA was determined by colorimetric assay at 260 nm, whereas the purity of RNA was determined by calculating the A260/280 nm ratio. The integrity of RNA was evaluated on 1% agarose gel electrophoresis with visualization of 18S and 28S rRNA bands. The complementary DNA (cDNA) was synthesized based on the Yakta Tajhiz Azma (YTA) cDNA synthesis kit (Cat No. YT 4500, Tehran, Iran). The mRNA expression was measured by ABI Step One Plus real-time PCR (Applied Biosystems, USA), using RealQ Plus 2× Master Mix Green High RO (Amplicon, Odense, Denmark) with specific primers for target genes (resistin, TNF-α, IL-6, IL-8, and ER-α), and glyceraldehyde-3-phosphate dehydrogenase (GAPDH) as an internal control. Relative expression software tool (REST, 2009 v2.0.13) was used for analysis based on the 2-ΔΔCt method. The specific primers for target genes were designed using Primer-Blast by The National Center for Biotechnology Information (NCBI) as shown in Table 1. The size and specificity of PCR products were determined by standard electrophoresis on a 1% agarose gel containing safe stain and melting curve analysis, respectively, as shown in Figure 1.

**Table 1:** The sequence of primers and PCR product length of target genes.

Genes	Primer length	Sequence (5ʹ →3ʹ)	PCR Product length (bp)
GAPDH	21	F-GGTCGGAGTCAACGGATTTGG	194
GAPDH	21	R-TGATGACAAGCTTCCCGTTCT	
Resistin	18	F-TACTTGCCCCCGAGGCTT	119
Resistin	18	R-CTCCGGTCCAGTCCATGC	
TNF- α	21	F-CCCATGTTGTAGCAAACCCTC	102
TNF-α	22	R-GCTGGTTATCTCTCAGCTCCAC	
IL-6	20	F-CATCCTCGACGGCATCTCAG	164
IL-6	20	R-TCACCAGGCAAGTCTCCTCA	
IL-8	20	F-CTTGGCAGCCTTCCTGATTT	68
IL-8	24	R-TTCTTTAGCACTCCTTGGCAAAAC	
ER-α	20	F-GGTGCCCTACTACCTGGAGA	85
ER –α	24	R-ATCTGAATTTGGCCTGTAGAATGC	

GAPDH: Glyceraldehyde 3-phosphate dehydrogenase; TNF-α: Tumor-necrosis factor-alpha; IL-6: Interleukin 6, IL-8: Interleukin 8; ER-α: Estrogen receptor alpha; F: Forward; R: Reverse.

### 2.4. Statistical analysis

Statistical analysis of the data was performed using a commercially available IBM Statistical Package for the Social Sciences (SPSS, Chicago, IL, USA) software. Quantitative data were presented as mean ± standard deviation (SD). The normal distribution of data was assessed by the Kolmogorov–Smirnov test. The comparison of normal distributed quantitative data between groups was done using ANOVA following Post hoc pairwise comparison Tukey test. For comparison of data that didn’t have normal distribution, the Kruskal–Wallis and Mann–Whitney U test was used. The association between gene expression and breast cancer was determined using logistic regression analysis, and the results are presented as the odds ratio (OR) [95% confidence intervals (CIs)]. Receiver operator characteristic (ROC) curve analysis and calculation of the area under the ROC curve (AUC) were carried out to assess the power of target genes in PBMCs to properly discriminate between breast cancer patients and controls. A two-sided P value of less than 0.05 was considered significant for all tests.

## 3.Results

### 3.1. Demographic and clinicopathological characteristics of the subjects

A total of 32 women with breast cancer were identified (median age, 45; range, 28–60 years), and 18 women without breast cancer were identified as healthy control (median age, 42; range, 36–53 years). Breast cancer patients were not significantly different from healthy controls in terms of age and BMI. The breast cancer patients were further divided into two subgroups based on with and without surgery. The results of cytopathology analysis for hormone-responsiveness biomarkers among 16 breast cancer patients without sugary indicated 12 (75 %) patients were with estrogen (ER)-positive, 13 (81.3%) patients with progesterone (PR)-positive, and 4 (25%) patients with HER2-positive; whereas, among 16 breast cancer patients with surgery, 13 (81.3%) patients were estrogen (ER)-positive, 10 (62.5) % patients were progesterone (PR)-positive, and 4 (25%) patients were HER2-positive (Table 2). Tumor grade was identified between grade 1 and grade III: grade I indicated well-differentiated tumors, grade II indicated moderately differentiated tumors, and grade III indicated poorly differentiated tumors. Based on the grading report, breast cancer patients without surgery showed the frequencies for grade I, II, and III were 9 (56.3%), 6 (37.5%), and 1 (6.3%), respectively; whereas, breast cancer patients with surgery indicated the frequencies of 4 (25%), 50% (8), and 25% (4), respectively for grade I, II, and III (Table 2).

**Table 2:** Characteristics of breast cancer patients.

Variable		Number	%
Age	≤ 45	17	53.1
	≥ 45	15	46.9
Tumor type	IDC	24	75.0
	ILC	8	25.0
Histological grade	Low (I)	13	40.6
	High (II/III)	19	59.4
ER	+	25	78.1
	-	7	21.9
PR	+	23	71.9
	-	9	28.1
HER2	+	8	25.0
	-	24	75.0

IDC: Invasive ductal carcinoma; ILC: Invasive lobular carcinoma; Grade I: well-differentiated tumor; Grade II: Moderately differentiated tumor; Grade III: poorly differentiated tumor; ER: estrogen receptor; PR: Progesterone receptor; HER2: Human epidermal growth factor receptor 2.

### 3.2. The mRNA expression of target genes in PBMCs

The mRNA expression of target genes was measured in the freshly ready RNA from PBMCs. Data are presented as fold change of gene expression measured in samples of breast cancer patients with and without surgery in comparison to controls (considered as a reference with fold expression = 1). 

**Figure 2:**
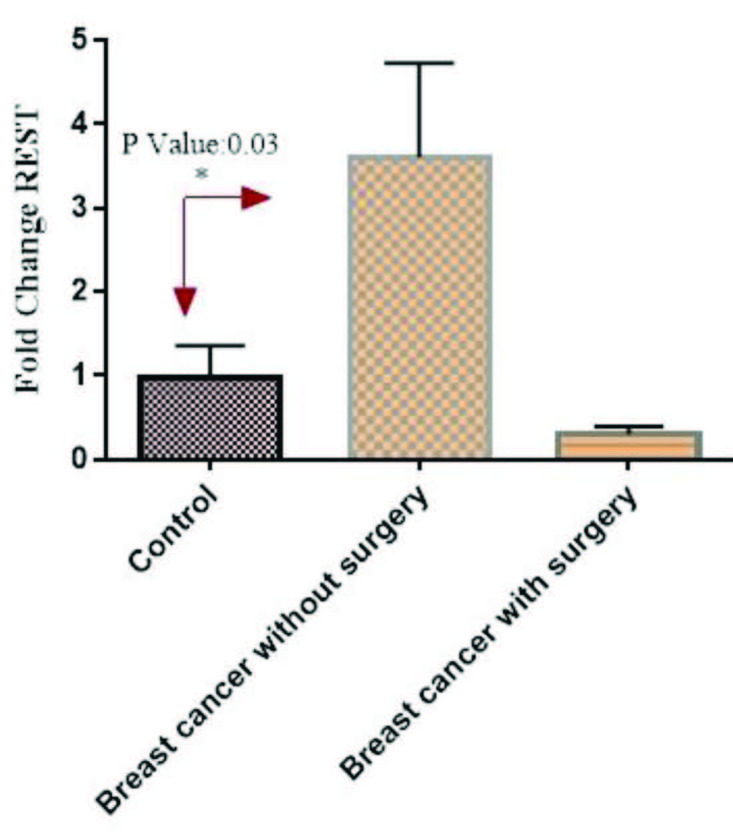
The mRNA expression of resistin in PBMCs from breast cancer patients and controls. Fold change expression of resistin in breast cancer patients compared to those from healthy controls determined using a quantitative reverse transcriptionpolymerase chain reaction (qRT-PCR). Fold change in mRNA level is shown under each bar. Error bars are standard errors of the means for resistin expression. Data are expressed as mean ± S.E.M. REST: Resistin.

**Figure 3:**
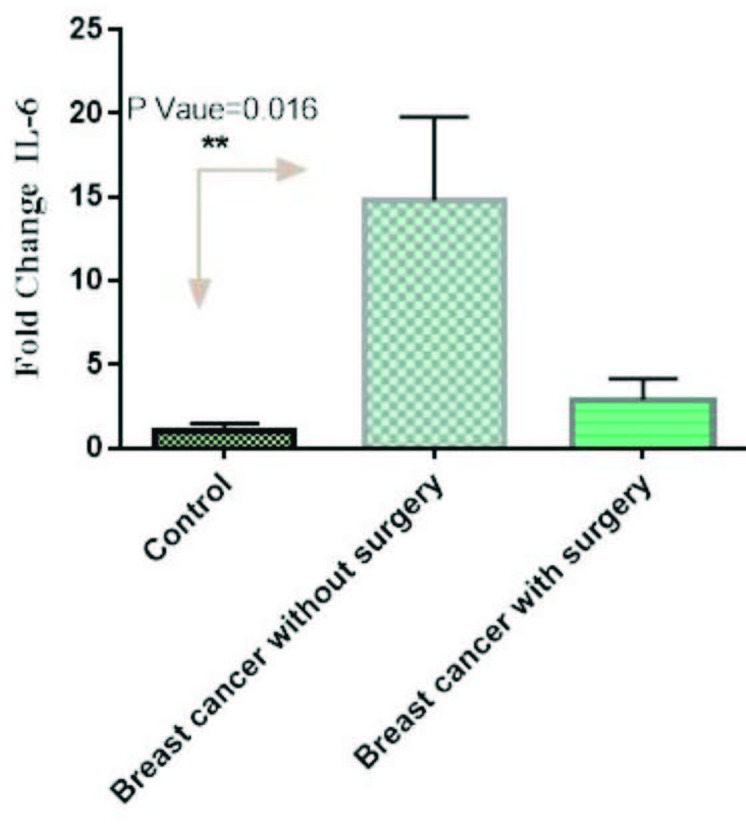
The mRNA expression of IL-6 in PBMCs from breast cancer patients and controls. Fold change expression of IL-6 in breast cancer patients compared to those from healthy controls determined using qRT-PCR. Fold change in mRNA level is shown under each bar. Error bars are standard errors of the means for IL-6 expression. Data are expressed as mean ±  S.E.M.

The mRNA expression of resistin was significantly high in patients without surgery in comparison to controls (p = 0.03). However, its mRNA expression was low in patients with surgery in comparison to controls, but not significant (p > 0.05) (Figure 2). Moreover, the mRNA expression of IL-6 was significantly high in patients without surgery in comparison to controls (p = 0.012). However, its mRNA expression was low in patients with surgery in comparison to controls, but not significant (p > 0.05) (Figure 3). On the other hand, its mRNA expression was significantly low in patients with lumpectomy subgroup in comparison to patients without surgery (data not shown here). 

**Figure 4:**
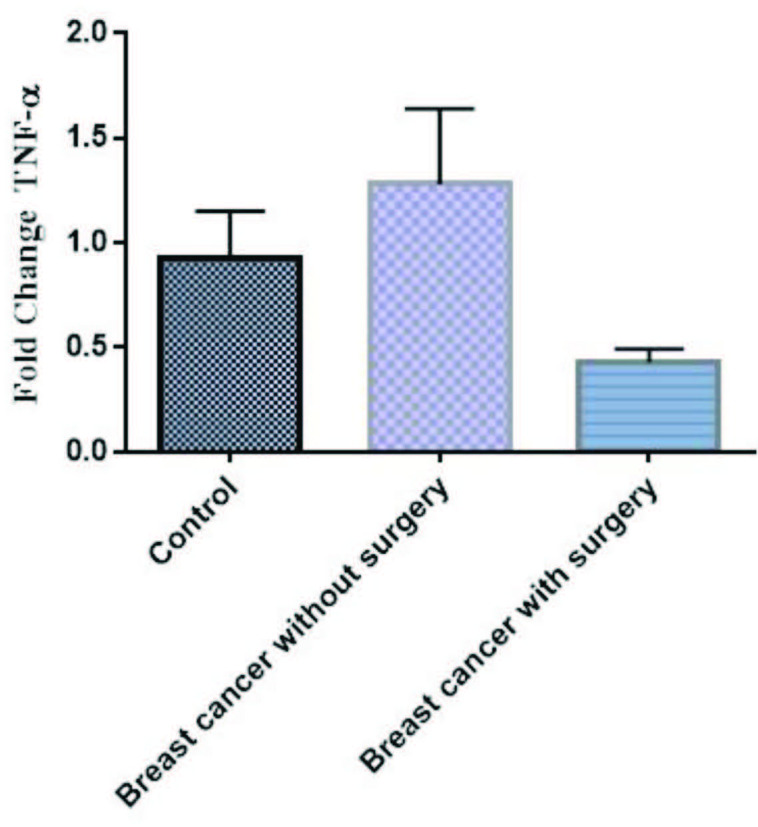
The mRNA expression of TNF-α in PBMCs from breast cancer patients and controls. Fold change expression of TNF-α in breast cancer patients compared to those from healthy controls determined using qRT-PCR. Fold change in mRNA level shown under each bar. Error bars are standard errors of the means for TNF-α expression. Data are expressed as mean ±  S.E.M

**Figure 5:**
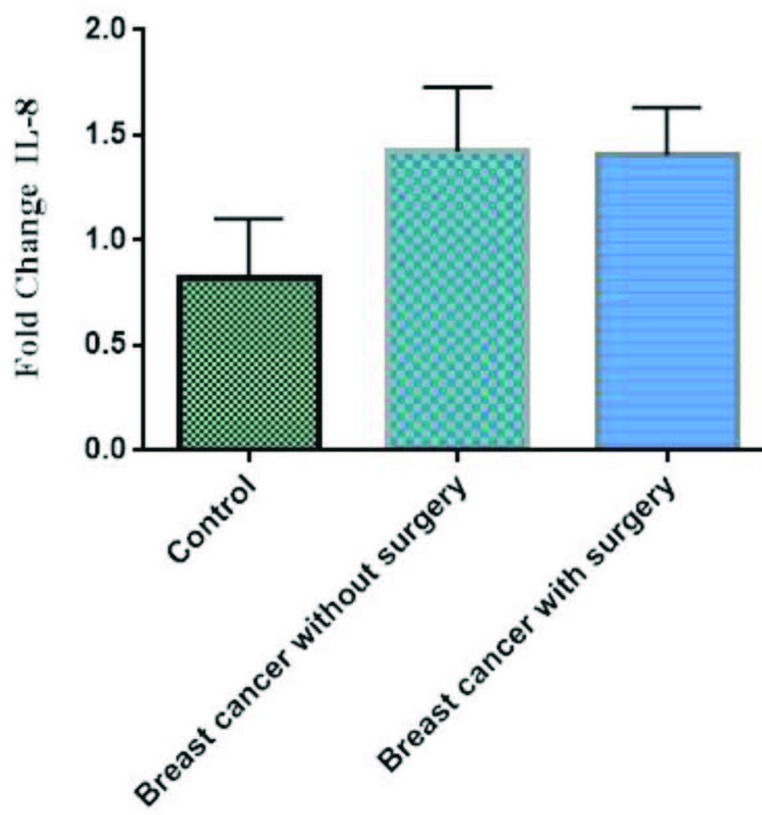
The mRNA expression of IL-8 in PBMCs from breast cancer patients and controls. Fold change expression of IL-8 in breast cancer patients compared to those from healthy controls determined by qRT-PCR. Fold change in mRNA level is shown under each bar. Error bars are standard errors of the means for IL-8 expression. Data are expressed as mean ±  S.E.M.

**Figure 6:**
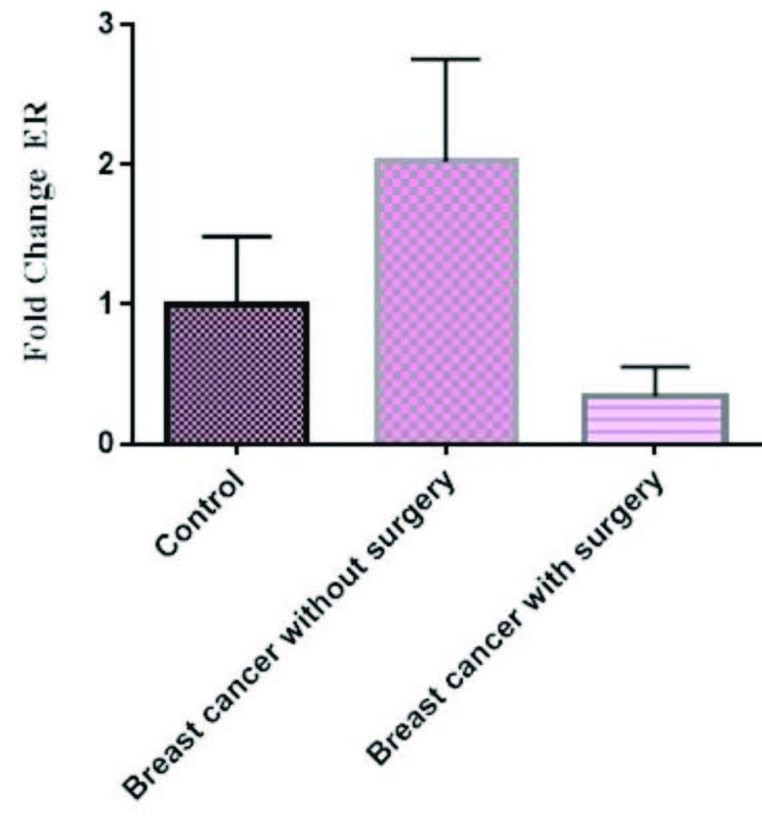
The mRNA expression of ER-α in PBMCs from breast cancer patients and controls. Fold change expression of ER-α in breast cancer patients compared to those from healthy controls determined using qRT-PCR. Fold change in mRNA level is shown under each bar. Error bars are standard errors of the means for ER-α expression. Data are expressed as mean ±  S.E.M.

The mRNA expression of TNF-α was high in patients without surgery in comparison to controls, but not significant (p > 0.05), whereas, its mRNA expression was low in patients with surgery in comparison to controls, but not significant (P> 0.05) (Figure 4). Also, our results indicated the mRNA expression of IL-8 was high in patients with and without surgery in comparison to controls, but not significant (p > 0.05) (Figures 5). Finally, the mRNA expression of ER-α was high in breast cancer patients without surgery in comparison to healthy controls, but not significant (Figure 6). However, its mRNA expression was significantly low in patients with mastectomy subgroup in comparison to patients without surgery (data not shown here).

Although, the mRNA expression of resistin in PBMCs of the population was not significantly associated with age (r = -0.228; p = 0.147) and BMI (r = -0.280; p = 0.165). However, the mRNA expression of resistin in PBMCs was significantly associated with the mRNA expression of IL-6 (0.412; p = 0.010), IL-8 (0.438; 0.011), TNF-α (0.490; p = 0.001), and ER-α (0.696; p < 0.001), respectively (data not shown here). Furthermore, logistic regression analysis indicated that none of the studied biomarkers were significantly associated with breast cancer (Table 3). Although, resistin’s diagnostic performance was not significant based on ROC curve analysis [0.680, 95% CI: 0.470–0.890, p = 0.110], the diagnostic performance of IL-6 was significant based on ROC curve analysis [0.825, 95% CI: 0.549–0.947, p = 0.030); thus, IL-6 might be considered as a breast cancer biomarker that reflecting an early and inflammatory stage of the disease (Table 4).

**Table 3:** The association between resistin, TNF-α, IL-6, IL-8, and ER-α expression and breast cancer.

Variable	Breast cancer/ controls	OR ( 95% CI)	p value
Resistin	32 / 18	1.140 (0.985-1.320)	0.078
TNF-α	32 / 18	1.169 (0.737-1.856)	0.176
IL-6	32 / 18	1.254 (0.936-1.681)	0.170
IL-8	32 / 18	1.082 (0.914-1.279)	0.359
ER-α	32 / 18	1.058 (0.885-1.264)	0.538

OR: Odds ratio; CI: Confidence interval; TNF-α: Tumor-necrosis factor-alpha; IL-6: Interleukin 6, IL-8: Interleukin 8; ER-α: Estrogen receptor alpha.

**Table 4:** Diagnostic value of PBMCs biomarkers for breast cancer patients.

Variable	AUC	OR (95% CI)	p-value
Resistin	0.680	0.470-0.890	0.110
IL-6	0.825	0.549-0.947	0.030
TNF-α	0.514	0.271-0.757	0.908
IL-8	0.522	0.254-0.793	0.870
ER-α	0.700	0.454-0.960	0.110

AUC: Area under the ROC curve; CI: Confidence interval; TNF-α: Tumor-necrosis factor-alpha; IL-6: Interleukin 6, IL-8: Interleukin 8; ER-α: Estrogen receptor alpha.

## 4. Discussion

High expression and increased serum level of harmful pro-inflammatory cytokines including resistin, TNF-α, IL-6, and IL-8 are associated with shorter survival and poor prognosis of breast cancer [2-5]. The use of tumor tissue samples for the analysis of gene expression has been reported in clinical practice [21]. Other studies showed that PBMCs can be used to develop gene expression-based tests for early detection of breast cancer and as a biomarker for identifying solid tumors at an early stage [22,23]. Generally, PBMCs are a valuable source of biomarkers in clinical and experimental studies because they can easily be obtained from patients and are readily available. To the best of our knowledge, there is no report regarding the association between resistin, TNF-α, IL-6, IL-8, and ER-α expression in PBMCs and breast cancer. We have demonstrated that the mRNA expression of resistin and ILl-6 in PBMCs of women with breast cancer was significantly high in comparison to women without breast cancer. 

Resistin, TNFa, IL-6, and IL-8 are major pro-inflammatory cytokines that are involved in systemic inflammation and acute-phase reactions. These cytokines have also been demonstrated that associated with the development of breast cancer [24]. Chronic inflammation not only has a major role in the development of cancer but also considered a condition that reflects the early pathologic changes in mammary cells and can be associated with the risk of breast cancer development [25,26]. A previous study has been demonstrated that resistin was highly expressed in serum and tumor tissue samples of patients with breast cancer [7]. Moreover, some studies found that the higher serum level of resistin was directly associated with more clinicopathological characteristics including tumor grade, tumor size, lymph node metastasis, inflammation, and metabolism. They suggested that resistin may be considered as a biomarker for the diagnosis and monitoring of endocrine therapy in patients with breast cancer [7,24]. 

Our results indicated that the mRNA expression of IL-6 significantly increased in PBMCs of breast cancer patients especially in patients without surgery in comparison to those with surgery and healthy controls. IL-6 and TNFa are two major cytokines that could involve in systemic inflammation and acute-phase reactions; also, their serum levels have been shown to increase in obese individuals and are consistently associated with the development of breast cancer [24,13]. Previous studies indicated that a high serum level of IL-6 and its soluble receptor has an independent prognostic value, and is associated with the extent and tumor grade of breast cancer [13,27]. We found that the mRNA expression of IL-6 significantly decreased in the patient with surgery in comparison to patients without surgery, which was also proved by the previous studies that showed serum level of IL-6 significantly decreased in patients after chemotherapy [28,29]. There is no report regarding the effect of surgery on the mRNA expression of IL-6; thus, we speculated that chemotherapy after surgery causes a reduction of IL-6 mRNA expression. Yokoe et al. in a study found that IL-6 level can be a predictive marker for recurrence of breast cancer [30]. On the other hand, IL-6 has been demonstrated to enhance breast cancer resistance to chemotherapy [31]; thus, targeting IL-6 could increase breast cancer sensitivity to chemotherapy [32]. Taken together, IL-6 not only has the potential to be a valuable predictive biomarker but also is a vital target for breast cancer treatment.

We found that the mRNA expression of TNF-α in PBMCs of breast cancer patients was higher than in control but not significant. However, its expression in patients with surgery (mastectomy) significantly decreased in comparison to those without surgery (data not shown here), which is consistent with the study conducted by Jabłonska et al. [33] who found that TNF-α level decreased after chemotherapy. We didn’t find any report regarding the association between surgery and TNF-α mRNA expression. Thus, we proposed that chemotherapy after mastectomy causes a reduction of its expression. TNF-α was detected as an important factor in the cytokine system and a major intermediate for cancer-associated inflammation [34]. TNF-α can induce tumor proliferation and can increase the invasions of breast cancer cells through the modulation of several metastasis-related genes [35,36]. Taken together, TNF-α can involve in the initiation and development of breast cancer [35].

We found the mRNA expression of IL-8 was high in breast cancer patients in comparison to controls but not significant. It has been demonstrated that serum level of IL-8 was significantly high and commonly associated with the enhanced clinical course, a higher tumor grade, and the presence of liver or lymph node involvement in high-grade breast cancer patients. [37]. The high expression of IL-8 in breast cancer cells with negative estrogen receptors can induce the high metastatic possibility of the cells [38]. 

Our results indicated that the mRNA expression of ER-α increased in breast cancer patients without surgery in comparison to controls but not significant; whereas, its expression significantly decreased in patients with mastectomy subgroup in comparison to patients without surgery (data not shown here). We didn’t find any report regarding the effect of surgery on the mRNA expression of ER-α in PBMCs, thus, we proposed that preoperative chemotherapy causes reduction of ER- α expression. This result is consistent with the result of the study of Lee et al. [39] who found that the expression of estrogen or progesterone receptor changed in 61% of breast cancers after preoperative chemotherapy. Preoperative chemotherapy is responsible for the reduction of the primary tumor and can facilitate surgical operation. However, its effect on the expression of the estrogen receptors is not completely understood. The assessment of estrogen and progesterone receptor expression not only is well-known management for breast cancer diagnosis but also is a predictive factor for the response of hormonal adjuvant therapy [40]. Taucher et al. [41] proposed that primary chemotherapy could significantly decrease estrogen receptor (ER) and progesterone receptor (PR) levels. They also observed that, in patients with preoperative chemotherapy, the size of ER-negative primary tumors significantly increased. Others didn’t find any effect of preoperative chemotherapy on the expression of estrogen receptors [42-44].

## 5. Conclusion

Our findings indicated that breast cancer especially in the early stage can affect the expression of certain genes in PBMCs. Measurement and analyze the expression of these genes have the potential to develop a PBMC-based gene-expression test for early detection of breast cancer. Moreover, according to these results, we suggest that the high expression of IL-6 in PBMCs may be considered as a new potential biomarker for the detection of breast cancer. However, further prospective and longitudinal studies are needed to evaluate whether inflammatory cytokines in PBMCs can be used as a predictive marker for the diagnosis and management of breast cancer.

## Informed consent

All individuals who participated in the study provided their written informed consent, and the Ethics Committee of the Ahvaz Jundishapur University of Medical Sciences approved the study (Ahvaz, Iran; approval no. IR.AJUMS.MEDICINE.REC.1398.005). The research protocol was in accordance with the principles of the 1964 Declaration of Helsinki with its later amendments.
